# In-hospital mortality and complications are increased in hematopoietic stem cell transplant recipients with myelodysplastic syndromes

**DOI:** 10.1007/s00277-026-07058-1

**Published:** 2026-06-12

**Authors:** Aniket Vijay Rao, Aditya Sanjeevi, Daniel Jose Idoate-Domench, Diya Jayanth Kamdar, Suraj Palavilayil, Abdullah Orakzai, Himal Kharel, Muhammad Hammad Sharif, Sujay Prabath Dronamraju

**Affiliations:** 1 Rochester Regional Health, Rochester, NY USA; 2 University of Rochester, Rochester, NY USA; 3 Blackpool Teaching Hospitals NHS, Blackpool, UK; 4 Rochester General Hospital, Rochester, NY USA; 5 Manipal Academy of Higher Education, Manipal, Karnataka India

## Abstract

Allogeneic hematopoietic stem cell transplantation (HSCT) remains the only curative therapy for patients with myelodysplastic syndromes (MDS). Despite evidence of comparable outcomes in selected older adults, disparities in HSCT utilization persist. This study analyzes trends, outcomes, and barriers such as age and insurance status associated with HSCT in MDS. We performed a retrospective analysis of the National Inpatient Sample (NIS) from 2016 to 2020 using ICD-10 codes to identify adult patients undergoing allogeneic HSCT. Patients were stratified according to MDS diagnosis. National estimates were calculated using discharge weights. Baseline characteristics and outcomes were compared using Pearson Chi-square and t-tests. Propensity score matching was used to adjust for potential confounders. Among 30,460 patients who underwent allogeneic HSCT, 4,980 (16.16%) had MDS. MDS patients were older (median age 62 vs. 49 years, p<0.001) and had a higher burden of comorbidities, including chronic lung disease, diabetes, and hypertension (p<0.001). They were more likely to be insured through Medicare (38.73% vs. 17.10%) and less likely to have private insurance (50.80% vs. 56.96%, p<0.001). From 2016 to 2020, the proportion of HSCT performed in patients with MDS increased from 13.20% to 17.32% before a slight decline in 2020. MDS patients experienced in-hospital mortality (aOR 1.33, 95% CI 1.14–1.55), mechanical ventilation (aOR 1.30), neutropenic fever (aOR 1.20), and acute GVHD (aOR 1.26), but lower Clostridium difficile infection (aOR 0.75). MDS patients undergoing allogeneic HSCT are older, carry greater comorbidity burdens, and have worse in-hospital outcomes. Insurance disparities and age may continue to represent barriers despite increasing utilization. Further research is needed to optimize selection and peri-transplant care.

## Keypoints

MDS patients undergoing HSCT had markedly higher in-hospital mortality, neutropenic fever, and respiratory failure than non-MDS patients.

National trends from 2016–2020 show MDS patients undergoing HSCT were older, had more comorbidities, and had longer hospital stays.

## Introduction

Myelodysplastic syndrome (MDS) is a clonal hematopoietic disorder characterized by ineffective hematopoeisis, cytopenias, and risk of progression to acute myeloid leukemia (AML) [1]. The only curative therapy is allogeneic hematopoietic stem cell transplantation (HSCT), with 35–40% of patients achieving long-term remission [2]. Despite advancements in understanding the molecular mechanisms of myelodysplastic syndromes (MDS), current therapies can extend survival but do not provide a cure [3]. As a result, allogeneic hematopoietic stem cell transplantation (HSCT) is increasingly utilized as a curative option [4]. This rise in HSCT procedures is partly due to the adoption of reduced-intensity (RI) conditioning regimens, which have expanded eligibility to patients with comorbidities or reduced fitness [5]. The growing use of unrelated or mismatched family donors has further contributed to the increased application of HSCT in MDS treatment [5]. The decision-making process for selecting suitable MDS candidates for HSCT involves evaluating both patient-related and disease-related factors, with transplant eligibility and outcomes influenced by factors such as age, comorbidities, insurance status, and race [6].

Historically, access to HSCT for MDS has been limited, particularly for older patients, due to concerns about toxicity and non-relapse mortality [7]. Reduced-intensity conditioning (RIC) regimens have expanded transplant eligibility, but real-world data on outcomes remain limited [8]. Insurance coverage also plays a crucial role; Medicare’s approval of HSCT for MDS in 2010 led to a fourfold increase in transplants for patients aged ≥ 65 [7]. Additionally, it is known that Black and Hispanic populations face lower donor availability and significant socioeconomic barriers, despite recent improvements in access from haploidentical transplantation [9–11]. Following transplant, in-hospital complications like neutropenic fever and acute graft versus host disease (AGVHD) are a concern for all patients undergoing hematopoietic stem cell transplant [12]. However, no studies have explored these complications in the subset of patients with MDS compared to patients without MDS undergoing HSCT. Among non-MDS patients undergoing HSCT, AML and acute lymphoblastic leukemia (ALL) are the most common, accounting for 35.1% and 22.4%, respectively [13]. 

Through this study, we evaluate nationwide trends and outcomes of allogeneic HSCT in MDS patients from 2016 to 2020, with an emphasis on in-hospital mortality, complications, and disparities related to age, insurance, and race in transplant eligibility. A direct comparison in outcomes was performed between the study population (MDS patients) and non-MDS patients receiving HSCTs.

## AIM

The primary objective of this study was to evaluate inpatient outcomes of allogeneic HSCT in MDS patients using a nationally representative dataset. We aimed to compare in-hospital mortality and major complications between MDS and non-MDS HSCT recipients while assessing the influence of age, race, and insurance status on transplant outcomes. Additionally, we examined disparities in access and trends in HSCT utilization for MDS from 2016 to 2020, with a focus on whether external factors, such as the COVID-19 pandemic, impacted transplant rates.

## Methods

### Study design

This retrospective cohort study analyzed the National Inpatient Sample (NIS) from 2016 to 2020, the largest all-payer inpatient database in the United States, maintained by the Healthcare Cost and Utilization Project (HCUP). The NIS captures a stratified 20% sample of all U.S. hospital discharges, allowing for national estimates through discharge weighting. Institutional review board approval was not required as the data is de-identified and publicly available.

Hospitalizations in which patients underwent allogeneic HSCT were identified using International Classification of Diseases, Tenth Revision (ICD-10) procedure codes. Patients with a primary or secondary diagnosis of MDS were classified as the MDS-HSCT cohort, while all other allogeneic transplant recipients were designated as the non-MDS-HSCT cohort. Patients undergoing autologous HSCT were excluded.

Patient demographics, including age, sex, race/ethnicity, and primary insurance payer, were extracted, along with hospital characteristics and comorbidities. The outcomes of interest included in-hospital mortality, neutropenic fever, Clostridioides difficile infection, respiratory failure requiring mechanical ventilation, acute kidney injury, venous thromboembolism, acute graft-versus-host disease, hospital length of stay, total hospitalization cost, and discharge disposition. ICD-10 codes for each diagnosis is listed in Table 1. (Table 1)

**Table 1:** ICD-10 Codes for Study Variables

Outcome	ICD-10 Code(s)
Allogeneic HSCT	30233G0, 30233T0, 30243G0, 30243T0
Myelodysplastic Syndrome (MDS)	D46.0, D46.1, D46.2, D46.4, D46.9
In-hospital Mortality	Discharge status of death
Neutropenic Fever	D70.1
Clostridioides difficile Infection	A04.7
Respiratory Failure (Ventilation)	J96.00, J96.01, J96.02, Z99.11, Z99.12
Acute Kidney Injury (AKI)	N17.0, N17.1, N17.2, N17.8, N17.9
Venous Thromboembolism (VTE)	I82.40, I82.42, I82.43, I26.90, I26.99
Acute Graft-versus-Host Disease	D89.810

### Statistical methods

Descriptive statistics compare baseline characteristics between MDS-HSCT and non-MDS-HSCT cohorts using Pearson’s chi-square test for categorical variables and Student’s t-test or Wilcoxon rank-sum test for continuous variables. Multivariable logistic regression estimated adjusted odds ratios (aORs) with 95% confidence intervals (CIs) for each outcome, adjusting for age, sex, race, insurance, and comorbidities. Propensity score matching (PSM) was performed for sensitivity analysis. All analyses accounted for the NIS survey design, incorporating stratification, clustering, and discharge weights to ensure national representativeness. Statistical significance was set at *P* < 0.05, and analyses were conducted using STATA 16 (StataCorp, College Station, TX).

## Results

### Patient characteristics

From 2016 to 2020, an estimated 30,460 patients underwent allogeneic HSCT in the United States, of whom 4,980 (16.16%) had MDS. The remaining patients who underwent HSCT for non-MDS related pathologies was estimated at 25,480 (83.8%). MDS-HSCT recipients were significantly older, with a median age of 62 years (IQR 56–68), compared to 49 years (IQR 25–62) for non-MDS patients (*P* < 0.001). Despite MDS typically being diagnosed around age 70, only 39% of MDS-HSCT patients were ≥ 65 years, reflecting selection bias favoring younger candidates. A slight male predominance was observed in both groups, though the proportion of female recipients was lower in MDS-HSCT (40.3% vs. 43.4%, *P* < 0.001). Racial disparities were marked between the two groups. White patients comprised 80.7% of MDS-HSCT recipients compared to 65.3% in non-MDS (*P* < 0.001), whereas Black (4.3% vs. 9.2%) and Hispanic (7.0% vs. 14.4%) patients were underrepresented, likely reflecting referral biases and donor availability. The remaining 8% were of other races (Asian/Pacific Islander, Native American, etc.), also slightly lower than in non-MDS (11.1%).

Insurance coverage varied significantly (*P* < 0.001), with Medicare insuring 38.7% of MDS-HSCT patients, more than double the rate in non-MDS (17.1%). This corroborates the older age distribution of the MDS group. Private insurance was the primary payer for 50.8% of MDS-HSCT recipients, while Medicaid coverage was markedly lower (6.6% vs. 18.2%). The proportion of uninsured/self-pay patients was also lower in MDS (2.9% vs. 5.2%). These findings suggest that lower-income younger patients (who would be on Medicaid or uninsured) are underrepresented in the MDS transplant cohort, whereas older patients covered by Medicare are overrepresented. This may point toward socioeconomic barriers in addition to the age factor for receiving HSCT in MDS.

MDS-HSCT patients had a higher burden of comorbidities, including chronic pulmonary disease (11.4% vs. 8.6%, *P* < 0.001), diabetes (15.7% vs. 10.1%, *P* < 0.001), hypertension (52.6% vs. 45.3%, *P* < 0.001), and obesity (11.7% vs. 9.9%, *P* = 0.0002). Comorbidities like congestive heart failure, chronic liver disease, and neurological disorders were similar. Table 2 lists the patient’s characteristics. Overall, the comorbidity profile indicates that despite having more co-existing illnesses, patients with significant comorbid burden were still being selected for HSCT in MDS. This may reflect careful pre-transplant evaluations where comorbid conditions were optimally managed, or it may suggest that by necessity (given the older age of MDS patients), transplants are being done in patients with higher HCT-Comorbidity Index scores. (Table 2)

**Table 2:** Summarizes the baseline demographics insurance, and comorbidities of the two groups. MDS patients undergoing allogeneic HSCT were typically older, predominantly White, and more likely covered by Medicare, with more medical comorbidities, compared to non-MDS transplant patients

Characteristic	Non-MDS HSCT (*N* = 25,840)	MDS HSCT (*N* = 4,980)	*P* value
Age, years – median (IQR)	49 (25–62)	62 (56–68)	< 0.001
Female sex, %	43.4%	40.3%	< 0.001
White, %	65.3%	80.7%	< 0.001
Black, %	9.2%	4.3%	-
Hispanic, %	14.4%	7.0%	-
Other, %	11.1%	8.0%	-
Medicare, %	17.1%	38.7%	< 0.001
Medicaid, %	18.2%	6.6%	-
Private Insurance, %	57.0%	50.8%	-
Self-pay/Uninsured, %	5.2%	2.9%	-
Chronic lung disease, %	8.6%	11.4%	< 0.001
Diabetes mellitus, %	10.1%	15.7%	< 0.001
Hypertension, %	45.3%	52.6%	< 0.001
Obesity, %	9.9%	11.7%	0.0002
Congestive heart failure, %	6.4%	6.7%	0.426
Neurologic disorder, %	6.6%	6.4%	0.726

### In-hospital outcomes

First, we examined unadjusted outcomes in absolute percentages. To account for the baseline differences between groups, we next examined adjusted outcomes using multivariable logistic regression. After adjusting for age, sex, race, insurance, and comorbidities, MDS diagnosis remained an independent risk factor for several adverse outcomes.

The in-hospital mortality rate in our cohort was relatively low in absolute terms. Among non-MDS HSCT patients, 4.5% died during the transplant hospitalization, whereas among MDS patients, the rate of death was 5.8%. This difference was statistically significant (4.47% vs. 5.82%, *P* < 0.001). On adjustment, MDS patients had 33% higher odds of in-hospital death compared to non-MDS HSCT patients (aOR 1.33, 95% confidence interval [CI] 1.14–1.55, *P* = 0.0002)​. It is noteworthy that this metric measures only in-hospital mortality. It is possible that some patients who survived discharge later died from transplant-related causes after discharge.

Several major complications showed significant differences between groups. Mechanical ventilation was required in 6.4% of MDS transplant patients vs. 5.2% of non-MDS patients, the odds of requiring mechanical ventilation about 30% higher in MDS patients (aOR 1.30, 95% CI 1.12–1.51, *P* = 0.0004)​. The need for ventilatory support often signifies severe respiratory failure, sepsis, or other critical illnesses during transplant. The higher rate in MDS patients suggests they experienced more frequent severe complications necessitating mechanical ventilation.

Neutropenic fever (fever/infection during neutropenia) was very common in both groups, but slightly more so in MDS patients: 41.5% vs. 38.2%. The adjusted odds of neutropenic fever remained significantly elevated as well (aOR 1.20, CI 1.12–1.28, *P* < 0.001)​. This implies that MDS patients had a higher incidence of infection requiring treatment during their transplant hospitalization. The actual rate of infection may be even higher.

These adjusted findings confirm that the higher mortality and infection/respiratory complications in MDS patients were not solely due to their older age or comorbidities, even after controlling for those factors, having MDS was associated with worse inpatient outcomes. This could point to factors intrinsic to MDS as a disease or its prior treatment (e.g. cumulative effects of prior therapies, poorer marrow function, etc.) that may predispose them to complications.

In contrast to the above, among patients undergoing HSCT, Clostridioides difficile infection (C. diff colitis) was less frequent in MDS patients, 6.6% in MDS vs. 10.6% of non-MDS patients. As expected from the unadjusted findings, MDS patients had significantly lower odds of C. difficile infection during the transplant admission (aOR 0.75, 95% CI 0.66–0.86, *P* < 0.001)​. Adjustment did not eliminate this difference, so it appears to be a robust and intriguing finding, as one might expect older, antibiotic-exposed patients to have more C. diff. We discuss possible explanations for this finding in the discussion.

The incidence of acute graft-versus-host disease (aGVHD) coded during the hospitalization was roughly 7% in both groups (7.13% MDS vs. 7.43% non-MDS), and this small difference was not statistically significant. Interestingly, after adjustment, a difference emerged in acute GVHD that was not apparent in unadjusted rates. MDS patients had significantly higher odds of acute GVHD (aOR 1.26, 95% CI 1.11–1.43, *P* = 0.0004) relative to non-MDS​. The increased risk of acute GVHD in MDS patients, despite their older age may reflect a higher proportion of mismatched or unrelated donor transplants in this cohort. It is important to consider that many cases of acute GVHD often manifest after initial discharge, so in-hospital coding underestimates true incidence. Here we capture only the early-onset or severe cases that required management before discharge or caused readmission during the initial hospitalization.

Two other complications, acute kidney injury (AKI) and venous thromboembolism (VTE), showed mixed results. AKI was common in both groups (23.09% in MDS vs. 20.10% in non-MDS. After adjusting for confounders, no statistically significant difference in odds of developing AKI was found (aOR 1.02 (0.94–1.10), *p* = 0.598). VTE (including pulmonary embolism) occurred in 5.02% of MDS patients versus 5.92% of non-MDS patients (*P* = 0.012). Adjustment showed reduced odds of developing VTE/PE, but it did not reach statistical significance (0.89 (0.77–1.03), *p* = 0.120).

Length of hospital stay for the transplant admission was prolonged in both cohorts, with a median of nearly 4 weeks. MDS patients had a median LOS of 26 days (IQR 22–33) versus 27 days (IQR 23–36) in non-MDS, and this difference was not statistically significant (*P* = 0.087). Hospital stay was long and comparable between groups. Consistent with the LOS, the median hospitalization cost was extremely high for both groups, reflecting the complexity of the transplant. The median total cost for non-MDS HSCT was $123,289 (IQR $82,485–$189,416) vs. $109,092 (IQR $75,942–$163,802) for MDS patients. While costs appeared somewhat lower for MDS (median difference ~$14,000), we did not have a reliable statistical comparison for cost in our data (due to inflation adjustments and hospital charge variations). These costs underscore that HSCT is an expensive resource-intense therapy​.

**Fig. 1:**
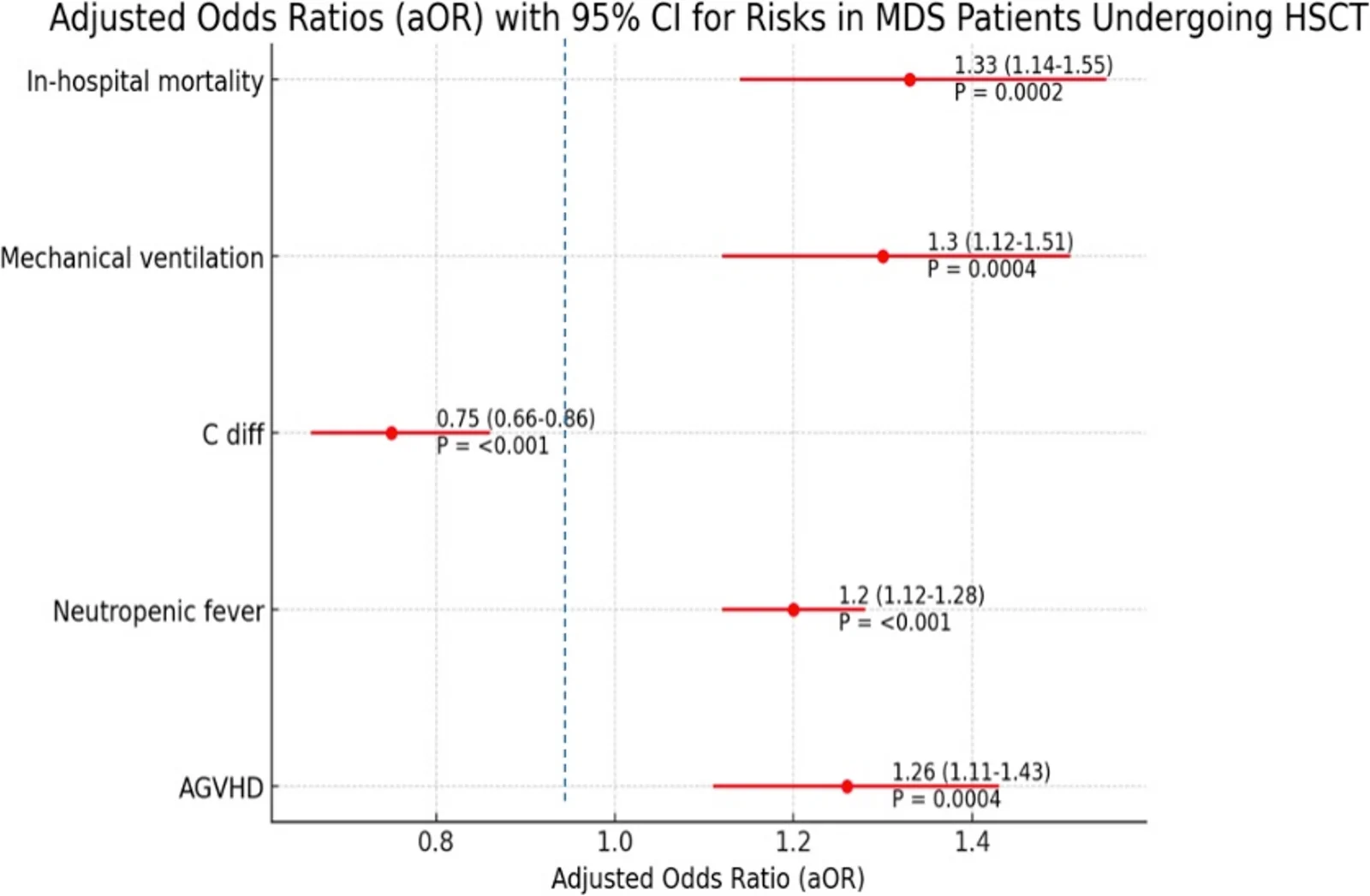
The propensity-matched analysis yielded findings consistent with the logistic regression. In the matched sample of 4,980 MDS vs 4,980 matched non-MDS patients (matched on age, sex, comorbidities, etc.), MDS patients still had significantly higher in-hospital mortality (5.8% vs 4.6%, P<0.01), higher mechanical ventilation and neutropenic fever rates, and lower C. diff rates, while GVHD was borderline higher. These reinforce that the observed outcome differences are not due to gross imbalances in baseline characteristics

Finally, we observed differences in discharge disposition. Among survivors, 65.8% of MDS patients were discharged home compared to 70.2% of non-MDS patients (*P* < 0.001 for overall disposition). A greater fraction of MDS patients required home health care services upon discharge (25.3% vs. 22.9%). The rates of transfer to a rehab or skilled nursing facility were low but slightly higher in MDS (2.6% vs. 2.0%). These patterns are not surprising given the older age of MDS patients – they were less likely to go straight home independently and more likely to need post-acute care support. This hints at greater frailty or caregiver needs in the older MDS cohort after transplant. Table 3 presents the unadjusted in-hospital outcomes of HSCT for MDS vs. non-MDS patients. (Fig. 1.) (Table 3.)

**Table 3:** In-hospital outcomes of HSCT for MDS vs. non-MDS patients

Outcome	Non-MDS HSCT (*N* = 25,840)	MDS HSCT (*N* = 4,980)	Adjusted Odds-Ratio (aOR) with 95% CI	*P* value
In-hospital mortality	4.47%	5.82%	1.33 (1.14–1.55)	0.0002
Mechanical ventilation	5.24%	6.43%	1.30 (1.12–1.51)	0.0004
AKI	20.10%	23.09%	1.02 (0.94–1.10)	0.598
C diff	10.62%	6.63%	0.75 (0.66–0.86)	< 0.001
Neutropenic fever	38.18	41.47%	1.20 (1.12–1.28)	< 0.001
VTE/PE	5.92%	5.02%	0.89 (0.77–1.03)	0.120
AGVHD	7.43%	7.13%	1.26 (1.11–1.43)	0.0004

### Trends in HSCT utilization for MDS (2016–2020)

**Fig. 2:**
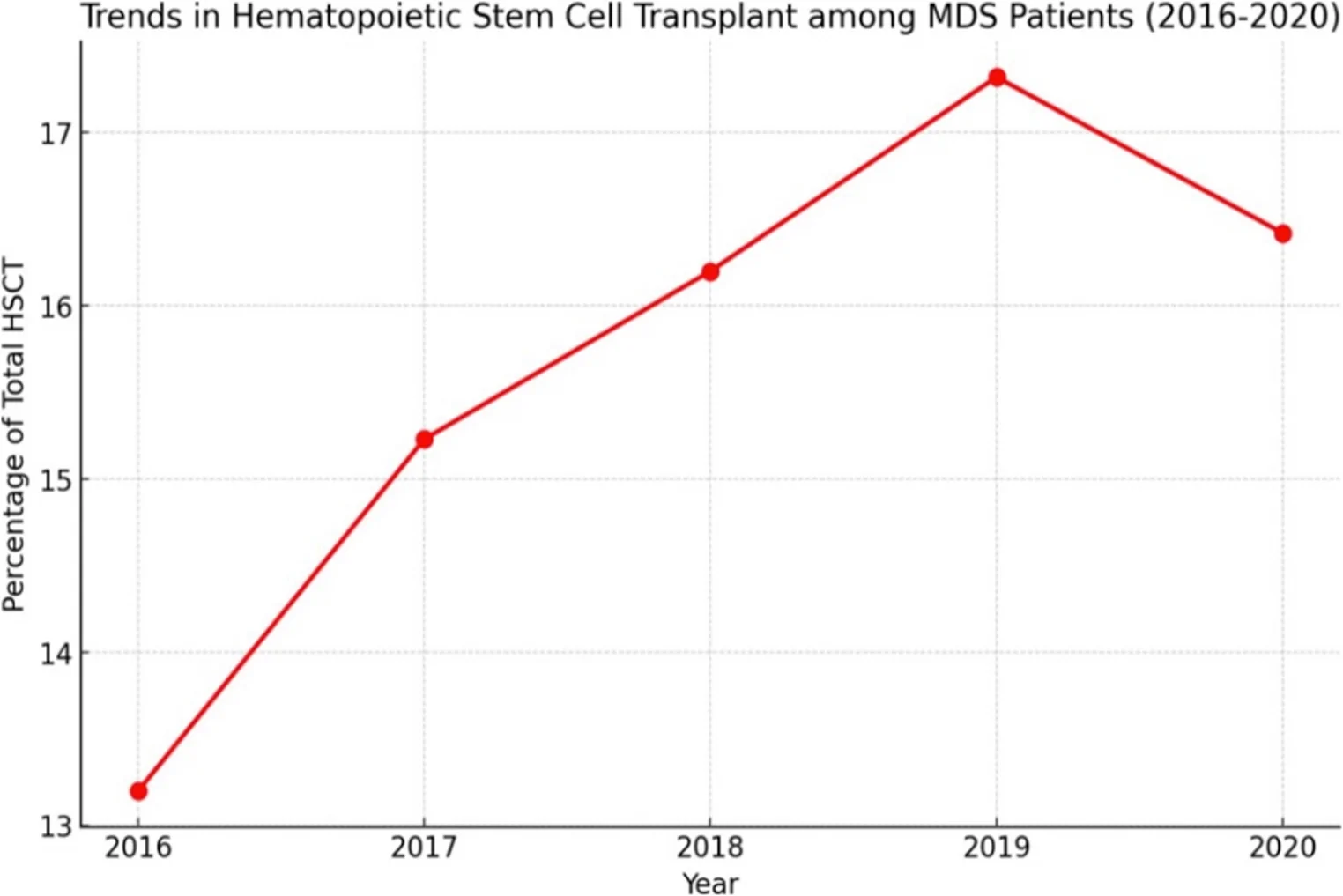
Trendsin allogeneic HSCT for MDS, 2016–2020. The graph denotes the proportion of transplants in that year that were for MDS. There was a steady increase in the utilization of HSCT for MDS from 2016 through 2019 (from 13.2% to 17.3% of all HSCTs), followed by a slight decline in 2020 (16.4%) likely due to the COVID-19 pandemic’s impact on transplant activities

Over the study period, we observed a steady increase in the number and proportion of HSCTs being done for MDS. In 2016, MDS patients accounted for only 13.2**%** of all allogeneic transplants (approximately 200 out of ~ 1,515 HSCT cases that year). This proportion rose each year, reaching 15.2% in 2017, 16.2% in 2018, and 17.3% in 2019 (where about 1,330 MDS transplants were done out of ~ 7,680 total HSCTs in 2019). Thus, by 2019, nearly one in six allogeneic transplants nationwide was for MDS, reflecting the growing utilization of HSCT as a therapeutic strategy in MDS. However, in 2020, we noted a slight dip: MDS comprised 16.4% of transplants (1,160 out of ~ 7,065 cases). In absolute terms, the number of MDS transplants dropped in 2020 compared to 2019 (1,160 vs. 1,330), as did the total HSCT volume. This decline coincided with the COVID-19 pandemic. It is possible that the pandemic interrupted or deferred some transplant plans in early 2020, especially affecting older or more elective transplant cases (many centers saw a temporary slowdown in transplants during the initial COVID surges). Indeed, 2020 was the only year in our series where the MDS transplant count and proportion fell from the previous year. Overall, the rising trend suggests improved acceptance of transplant for MDS, potentially due to accumulating evidence of efficacy in older patients, more referrals of high-risk MDS earlier in the disease course, and expanded donor options (e.g., use of haploidentical donors in recent years).The plateau/drop in 2020 is an outlier, likely reflecting external factors rather than a reversal of the trend. (Fig. 2.)

In summary, our results demonstrate that MDS patients represent a growing segment of allogeneic HSCT recipients, although they tend to be younger than the overall MDS population and are predominantly insured through Medicare or private insurance. In-hospital outcomes for MDS transplant patients are worse in certain respects (higher mortality, more complications) even after adjusting for baseline differences. Below, we discuss the implications of these findings, especially regarding the roles of age, insurance, and race in transplant outcomes and access for MDS.

## Discussion

Myelodysplastic syndromes (MDS) are clonal disorders of hematopoietic stem cells, characterized by progressive cytopenia and a significant risk of evolving into acute myeloid leukemia (AML) [14]. In 2001, the International Classification of Diseases for Oncology, Third Edition (ICD-O-3) officially classified MDS as a malignancy. Primarily affecting older adults, the median age of diagnosis is 77 years [15]. Current treatment approaches focus on supportive care and include erythropoiesis-stimulating agents (ESAs), lenalidomide, hypomethylating therapy, luspatercept, and immunosuppressive agents. However, the only potential curative treatment remains allogeneic hematopoietic cell transplantation, which has a 35–40% success rate [8, 9]. However, transplant eligibility and outcomes are influenced by factors such as age, comorbidities, insurance status, and race [16].

In this study, we conducted a nationwide analysis of allogeneic hematopoietic stem cell transplantation (HSCT) for patients with myelodysplastic syndrome (MDS) versus those without MDS. We found that MDS patients undergoing HSCT are significantly older and have a higher comorbidity burden compared to non-MDS transplant recipients. They also experience higher in-hospital mortality and complications, even after adjustment for baseline differences. Our findings highlight key disparities in age, insurance status, and race that affect both access to and outcomes of HSCT in MDS patients.

Despite being a disease predominantly affecting individuals aged 65–70, the median age of MDS patients undergoing HSCT was 62 years, indicating that older patients remain underrepresented in transplant cohorts. Many factors contribute to this selection bias, including concerns over transplant-related toxicity and provider hesitancy to refer elderly patients for HSCT [7, 17]. However, studies have shown that carefully selected elderly patients (65–75 years) can achieve similar long-term outcomes to younger patients if comorbidities are well-managed [9, 10]. The expansion of reduced-intensity conditioning (RIC) regimens has allowed more older adults to undergo HSCT, but significant gaps remain in translating these advances into broader clinical practice [18].

Insurance coverage is another critical factor influencing access to HSCT. Before 2010, Medicare did not cover HSCT for MDS outside of clinical trials, limiting transplant access for older patients. After Medicare’s policy change, transplants for MDS in patients aged ≥ 65 quadrupled, emphasizing the role of insurance in access to curative therapy [19]. Our study demonstrates that Medicaid and uninsured patients were significantly underrepresented among MDS transplant recipients, suggesting that socioeconomic barriers persist. Medicaid patients may face difficulties in securing transplant evaluations, traveling to specialized centers, or obtaining post-transplant support [19]. These findings highlight the need for policy interventions to improve financial accessibility for younger, lower-income patients with MDS.

Racial disparities in HSCT for MDS are also pronounced. Our study corroborates previously published data that most transplant recipients were White. In contrast, Black and Hispanic patients were underrepresented [11]. The major barrier for minority patients is lower donor availability, particularly among African Americans, who have only about a 16% chance of finding a fully matched unrelated donor, compared to ~ 75% for White patients. A postulated explanation for this phenomenon relates to greater HLA diversity in the African American population. The increasing use of haploidentical transplantation has helped address this issue, improving access for minority patients, but further progress is needed [20]. African Americans also see lower representation in national donor registries [21]. Additionally, systemic barriers such as lower referral rates, socioeconomic challenges, and provider biases may contribute to these disparities [22]. Expanding minority donor recruitment and haploidentical HSCT programs could help bridge these gaps.

Regarding in-hospital outcomes, our study found that MDS patients had a significantly higher risk of in-hospital mortality, along with increased rates of neutropenic fever, mechanical ventilation, and acute GVHD compared to non-MDS transplant recipients. The higher mortality risk persisted after adjustment, suggesting intrinsic disease-related factors contribute to worse outcomes. Possible explanations include long-standing cytopenia, iron overload from transfusions, and cumulative toxicity from pre-transplant therapies [23]. Pre-transplant ferritin, a surrogate marker for iron overload, is associated with increased non-relapse mortality and inferior transplant outcomes in patients with MDS, but could not be assessed in our analysis [24].

One notable finding was the lower incidence of Clostridioides difficile infection among MDS transplant recipients compared with non-MDS recipients. This may reflect differences in prior antibiotic exposure, with patients diagnosed with acute leukemia, for example, often receiving intensive chemotherapy and prolonged inpatient care before HSCT, increasing their risk of C. difficile infection [25]. Emerging data have demonstrated that alterations in intestinal microbiome composition may be strongly associated with transplant-related outcomes, such as infection, graft-versus-host disease, and overall survival. Reduced microbial diversity and antibiotic-induced dysbiosis have been linked to adverse outcomes following stem cell transplant [26, 27]. Although microbiome composition and antibiotic utilization patterns cannot be assessed using the NIS, prospective studies incorporating microbiome profiling are warranted to explore relationships between microbial ecology and transplant outcomes.

Finally, we observed a steady increase in HSCT utilization for MDS from 2016 to 2019, rising from 13.2% to 17.3% of all allogeneic transplants. This trend reflects greater acceptance of transplants for MDS, likely driven by improved conditioning regimens, expanded donor options, and a better understanding of risk-stratified transplant decision-making [28, 29]. However, a drop in transplants in 2020 coincided with the COVID-19 pandemic. Other studies have demonstrated a 3.5% decrease in unrelated donors’ HSCTs globally [30]. Reports also suggest that Allogeneic HCT for the myeloproliferative syndromes decreased for MDS by 4.3%. Part of this was thought to be contributed by lockdowns, which were noted to have disturbed the supply chains and limited access to critical care facilities such as ICU wards [30]. Future studies should assess whether transplant rates rebounded post-pandemic.

This study has several limitations inherent to the use of an administrative database. The National Inpatient Sample does not provide detailed cytogenetic, molecular, or laboratory-level data. As a result, stratification by contemporary MDS subgroups, including complex karyotype, granular blast percentage categories, or newly defined entities in the WHO 2022 classification, such as MDS-F, could not be performed. In addition, laboratory parameters, including ferritin, C-reactive protein, and lactate dehydrogenase, are not captured. The dataset also lacks information regarding transfusion burden, iron overload status, iron chelation therapy, antibiotic exposure patterns, and microbiome composition. These factors may influence transplant-related morbidity and mortality, but could not be assessed in the present analysis. Prospective or registry-based studies incorporating molecular risk stratification, laboratory markers, and microbiome profiling are needed to better define predictors of transplant outcomes in patients with MDS.

## Conclusion

Allogeneic hematopoietic stem cell transplantation (HSCT) remains the only curative option for myelodysplastic syndromes (MDS), yet significant disparities persist in access and outcomes. Between 2016 and 2020, MDS accounted for 16% of all allogeneic transplants, with recipients being younger than the general MDS population, mostly insured, and predominantly White. Despite advances in transplant techniques, MDS patients experienced higher in-hospital mortality and complication rates, even after adjusting for age and comorbidities, confirming they are a high-risk transplant population.

Barriers to HSCT persist for older adults, racial minorities, and socioeconomically disadvantaged patients. While reduced-intensity conditioning and haploidentical transplantation have improved access, Medicaid recipients and uninsured patients remain underrepresented. Expanding frailty screening, financial support programs, and targeted donor recruitment could help bridge these gaps. Since fit older patients can achieve comparable outcomes to younger recipients, age alone should not preclude HSCT referral.

MDS patients undergoing HSCT face higher risks of neutropenic fever, respiratory failure, and acute GVHD, highlighting the need for optimized GVHD prophylaxis, infection prevention, and post-transplant monitoring. Policy changes ensuring equitable transplant access and financial support for underinsured patients are also necessary to address socioeconomic disparities.

In summary, allogeneic HSCT for MDS is increasing, but disparities in age, insurance, and race continue to influence access and outcomes. Efforts to optimize peri-transplant care, expand eligibility, and reduce inequities are essential to improving long-term survival and ensuring equitable access to curative therapy for all eligible MDS patients.

These findings should be interpreted in the context of the inherent limitations of an administrative database, including the absence of molecular, laboratory, and treatment-level data. Prospective studies incorporating detailed risk stratification and biologic markers are needed to further refine outcome prediction in MDS patients undergoing HSCT.

## Data Availability

The data analyzed in this study are publicly available from the Healthcare Cost and Utilization Project (HCUP) National Inpatient Sample (NIS) for the years 2016–2020. Access is available through registration and purchase on HCUP. All data processing and code used to generate the results are available from the corresponding author upon reasonable request.
